# Effects of vitamin D on insulin resistance and myosteatosis in diet-induced obese mice

**DOI:** 10.1371/journal.pone.0189707

**Published:** 2018-01-17

**Authors:** Elisa Benetti, Raffaella Mastrocola, Fausto Chiazza, Debora Nigro, Giuseppe D’Antona, Valentina Bordano, Roberto Fantozzi, Manuela Aragno, Massimo Collino, Marco Alessandro Minetto

**Affiliations:** 1 Dipartimento di Scienza e Tecnologia del Farmaco, University of Turin, Turin, Italy; 2 Department of Clinical and Biological Sciences, University of Turin, Turin, Italy; 3 Department of Public Health, Molecular and Forensic Medicine, and Sport Medicine Centre Voghera, University of Pavia, Pavia, Italy; 4 Division of Endocrinology, Diabetology and Metabolism, Department of Medical Sciences, University of Turin, Turin, Italy; 5 Division of Physical Medicine and Rehabilitation, Department of Surgical Sciences, University of Turin, Turin, Italy; Universidade do Estado do Rio de Janeiro, BRAZIL

## Abstract

Epidemiological studies pointed out to a strong association between vitamin D deficiency and type 2 diabetes prevalence. However, the role of vitamin D supplementation in the skeletal muscle, a tissue that play a crucial role in the maintenance of glucose homeostasis, has been scarcely investigated so far. On this basis, this study aimed to evaluate the effect of vitamin D supplementation in a murine model of diet-induced insulin resistance with particular attention to the effects evoked on the skeletal muscle. Male C57BL/6J mice (n = 40) were fed with a control or a High Fat-High Sugar (HFHS) diet for 4 months. Subsets of animals were treated for 2 months with vitamin D (7 μg·kg-1, i.p. three times/week). HFHS diet induced body weight increase, hyperglycemia and impaired glucose tolerance. HFHS animals showed an impaired insulin signaling and a marked fat accumulation in the skeletal muscle. Vitamin D reduced body weight and improved systemic glucose tolerance. In addition, vitamin D restored the impaired muscle insulin signaling and reverted myosteatosis evoked by the diet. These effects were associated to decreased activation of NF-κB and lower levels of TNF-alpha. Consistently, a significantly decreased activation of the SCAP/SREBP lipogenic pathway and lower levels of CML protein adducts and RAGE expression were observed in skeletal muscle of animals treated with vitamin D.

Collectively, these data indicate that vitamin D-induced selective inhibition of signaling pathways (including NF-κB, SCAP/SREBP and CML/RAGE cascades) within the skeletal muscle significantly contributed to the beneficial effects of vitamin D supplementation against diet-induced metabolic derangements.

## Introduction

Vitamin D is a prohormon with multiple functions that extend beyond the regulation of the intestinal calcium absorption. In recent years, a large number of studies investigated the association between vitamin D status (and/or supplementation) and health [1, 2].

In particular, epidemiological studies pointed out to a strong association between vitamin D deficiency and type 2 diabetes (T2DM) prevalence [3–6]. Consistently, serum 25-hydroxyvitamin D concentrations were negatively associated with body mass index [7, 8], insulin resistance and β-cell dysfunction [9–11].

Moreover, results from preclinical studies showed that vitamin D (or calcitriol) administration reduced the levels of blood glucose and improved insulin sensitivity in diabetic mice [12], attenuated the fibrosis and the increased expression of the receptor for advanced glycation end products (RAGE) in hearts of diabetic rats, and improved high fat diet-induced metabolic syndrome and reduced hepatic steatosis in rats [13, 14]. We previously found that mice fed an high-fat-high sugar diet developed intra-muscular accumulation of both fat and advanced glycations end products (AGEs) in association with metabolic abnormalities [15]. Although the factors leading to accumulation of intra- and intermuscular fat, a phenomenon known as myosteatosis (or ectopic skeletal muscle adiposity), are not well understood, we suggested that the accumulation of AGEs is the possible molecular mechanism linking the impairment of insulin sensitivity to activation of lipogenesis (leading to myosteatosis) and to changes in muscle fiber size and composition that occur in diet-induced obese mouse skeletal muscle [15, 16]. Consistently, recent evidence from human studies also indicated that myosteatosis is linked to reduced insulin sensitivity and loss of muscle performance [17, 18]. Therefore, it may be hypothesized that the improvement of skeletal muscle insulin sensitivity induced by vitamin D can be paralleled by a reduction of diet-induced myosteatosis. The aim of the present study was to investigate whether vitamin D administration improves skeletal muscle insulin sensitivity and metabolic profile of diet-induced obese mice and whether these improvements could be associated to reduction of myosteatosis.

## Materials and methods

### Animals and experimental procedures

Four-week-old male C57BL/6J mice (n = 40, provided by Charles River, Lecco, Italy) were housed in a controlled environment at 25±2°C with alternating 12-h light and dark cycles and fed normal diet during a 1 week adaptation period. The animals were then randomly allocated to two experimental groups: mice fed a control diet or an High Fat-High Sugar (HFHS) diet (ssniff Spezialdiäten GmbH, Ferdinand-Gabriel-Weg, Germany) for 16 weeks. The HFHS diet contained 45% kcal fat (lard and soybean oil), 20% protein (casein), 35% carbohydrate. The diet sugar component, a well known source of AGEs, was represented by fructose 55% and glucose 45%. Diet compositions are detailed in the Supplementary Materials (S1 Table). The animal protocol (n. DGSAF0021253-A-07/11/2013) was approved by the local ‘Animal Use and Care Committee’ (Comitato di Bioetica di Ateneo, University of Turin) and it was in accordance with the European Directive 2010/63/EU on the protection of animals used for scientific purposes.

### Vitamin D administration

After the initial period of 8 weeks of dietary manipulation (control diet or HFHS diet), the animals were randomly allocated to four groups for the following 8 weeks: Control group (Control, n = 10), Control group+vitamin D (Control+vit D, n = 6), HFHS group (HFHS, n = 10), HFHS+vitamin D (HFHS+vit D, n = 14).

The active form of Vitamin D (1,25-dihydroxycholecalciferol) was administered at the dose of 7 μg/kg i.p. three times/week. The dose was chosen according to a previous studies demonstrating that vitamin D supplementation had a positive effect on glucose homeostasis and reduced diet-induced hepatic steatosis [12, 19].

### Oral glucose tolerance test (OGTT)

The day of the sacrifice, the OGTT was performed after a fasting period of 16 hours. Once before glucose administration (2 g/kg by oral gavage) and 15, 30, 60 and 120 minutes afterwards, blood samples were obtained from the saphenous vein puncture, and glucose concentration was determined with a conventional Glucometer (Glucomen LX Plus, A. Menarini Diagnostics, Florence, Italy).

### Procedures and analyses

Body weight was recorded weekly. At the end of the study (week 16), after 16 h fasting period, the mice were anesthetized using isoflurane via an anesthesia machine (IsoFlo, Abbott Laboratories) and sacrificed by cardiac puncture/exsanguination. Blood was collected for biochemical analyses and the gastrocnemii were rapidly removed, frozen in N_2_ and stored at −80°C. Plasma serum 25-OH vitamin D level was measured using enzyme-linked immunosorbent assay (ELISA) kit (ab213966 25-OH Vitamin D ELISA kit, Abcam, Cambridge, UK). Glycemia was measured using the GlucoMen LX kit. Plasma lipid profile was determined by measuring the content of triglycerides (TGs), total cholesterol, high-density-lipoprotein (HDL) cholesterol by using commercial reagent kits (Hospitex diagnostics, Florence, Italy). Low-density-lipoprotein (LDL) cholesterol was determined by the following calculation [LDL = total cholesterol–(HDL + TG/5)]. Plasma insulin was measured using enzyme-linked immunosorbent assay (ELISA) kits (Mercodia Insulin ELISA). The homeostasis model assessment of insulin resistance (HOMA-IR) was determined by the following calculation [HOMA-IR = (fasting plasma glucose [mmol/l] * fasting plasma insulin [μUI/ml]) /22.5][20].

### Tissue extracts and analyses

Gastrocnemious extracts were prepared as previously described [21]. Briefly, tissues were homogenized and centrifuged. Supernatants were removed and the protein content was determined using a BCA protein assay following the manufacturer’s instructions (Pierce Biotechnology Inc. Rockford, IL, USA).

About 50 μg of total proteins were loaded for Western blot experiments as previously described [21, 22]. After blocking, the PVDF membranes were incubated at 4°C overnight with antibodies against ph- Insulin Receptor Substrate 1 (ph-IRS-1, 1:1000, Cell Signaling Technology, #2381), IRS-1 (1:1000, Cell Signaling Technology, #3196), ph- Protein kinase B, also referred to as Akt (1:1000, Cell Signaling Technology, #4051), Akt (1:1000, Cell Signaling Technology, #9272), ph- Glycogen Synthase Kinase-3β (ph-GSK-3β, 1:1000, Cell Signaling Technology, #9322), GSK-3β (1:1000, Cell Signaling Technology, #9315), Nuclear Factor Kappa-light-chain-enhancer of activated B cells (NF-κB) p65 (1:1000, Cell Signaling Technology, #8242), Sterol Regulatory Element-Binding Protein (SREBP)-1c (1:500, Abcam Cambridge, UK, ab44153), SREBP Cleavage-Activating Protein (SCAP, 1:500, Abcam, ab125186), CarboxyMethyl Lysine (CML, R&D System, Minneapolis, MN, USA, #MAB3247), Receptor for Advanced Glycation End products (RAGE, 1:500, Abcam, ab78022). The membranes were stripped and incubated with GlycerAldehyde 3-Phosphate DeHydrogenase antibody (GAPDH, 1:1000, Abcam ab9485) or histone H3 antibody (1:200, Santa Cruz Biotechnology, sc10809) to assess gel-loading homogeneity. Proteins of interest were detected with horseradish peroxidase-conjugated secondary antibody (1:5000, Cell Signaling Technology) for 1 h at room Temperature. The results were quantified using ImageJ software.

Gastrocnemious TNF-alpha levels were measured by ELISA (Quantikine ELISA Kit, R&D Systems).

Gastrocnemious intramyocellular lipid accumulation was evaluated by Oil Red O staining on 10 μm cryostatic sections. Stained tissues were viewed under an Olympus Bx4I microscope (40x magnification) with an AxioCamMR5 photographic attachment (Zeiss, Göttingen, Germany).

Unless otherwise stated, all compounds were purchased from the Sigma-Aldrich Company Ltd. (St. Louis, Missouri, USA). PVDF was from Millipore Corporation (Bedford, MA, USA). Primary and secondary antibodies were from Cell-Signaling Technology (Beverly, MA, USA) and Luminol ECL from PerkinElmer (Waltham, MA, USA).

### Statistical analysis

One-way or Two-way analysis of variance with Bonferroni's post-hoc test were adopted for analysis of normally distributed data and the results are expressed as mean±S.E.M. The Kruskal–Wallis Anova followed by Dunn’s post hoc test were adopted for analysis of non-normally distributed data and results are expressed by median and interquartile range (and represented as Box and Whiskers plots). The analyses were performed by using the GraphPad Prism version 5.0 for Windows (GraphPad Software, San Diego, California, USA), and p values <0.05 were considered as significant.

## Results

### Effects of diet manipulation and 1,25 (OH)_2_ vitamin D supplementation on mice body weight

**Fig 1:**
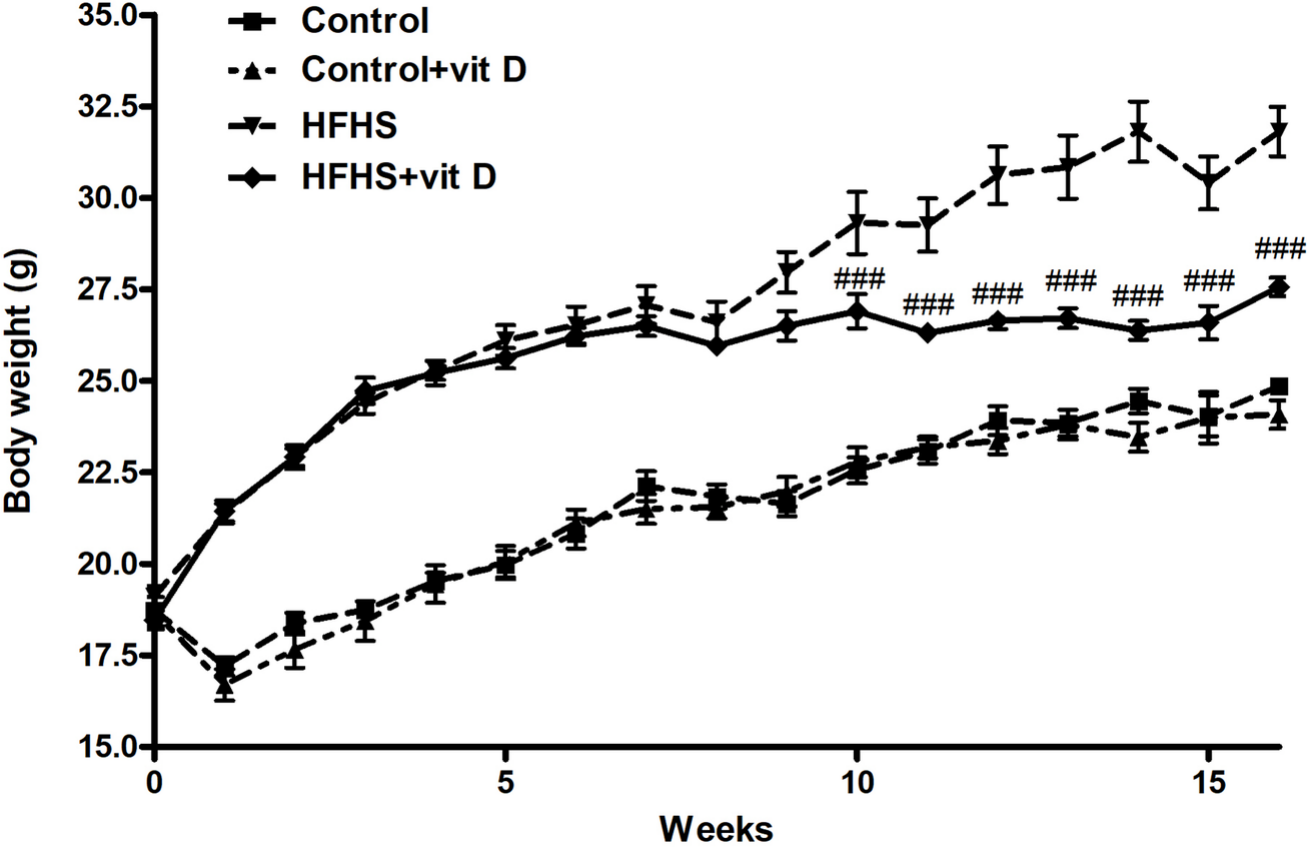
Effects of diet manipulation and 1,25 (OH)_2_ vitamin D supplementation on mice body weight. Values are means ± S.E.M. of 6–14 animals per group. Statistical analysis was performed with Two-way analysis of variance with Bonferroni's post-hoc test. ^###^p<0.001 *vs* HFHS. p<0.001 HFHS groups *vs* Control groups from week 1, symbol are not reported in the figure.

As shown in Fig 1, the HFHS diet dramatically increased mice body weight, with a significant effect already after the first week of diet manipulation (p<0.001 HFHS *vs* control from week 1 to week 16, the relative symbols are not reported in the Figure).

Vitamin D supplementation (weeks 8–16) did not affect the average body weight of control animals (average body weight at week 16: 24.85±0.24 g for Control group and 24.08±0.48 g for Control+vit D group), but was associated with a significant body weight reduction of animals fed HSHS diet (average body weight at week 16: 31.82±1.04 g for HFHS group and 27.57±0.56 g for Control+vit D group; p<0.001 *vs* HFHS). This effect became statistically significant after 2 weeks of vitamin D supplementation (at the week 10) and is well evident when the body weight gain during the treatment period (weeks 8–16) is evaluated (∆BW_weeks 8–16_ = Control 3.33±0.18 g; Control+vit D 2.4±0.31 g; HFHS 5.16±0.51 g; HFHS+vit D 1.51 ±0.16 g. HFHS+vit D *vs* HFHS: p<0.001).

Moreover, the administration of vitamin D significantly blunted the diet-induced increase of gastrocnemious weight (Control 0.13±0.004 g; Control+vit D 0.13±0.008 g; HFHS 0.16±0.004 g; HFHS+vit D 0.14 ±0.002g. HFHS+vit D *vs* HFHS: p<0.001).

By analyzing the average food intake (g/die) no differences were observed among the groups by considering the full length of the experimental protocol (weeks 1–16); whereas by focusing on the vitamin D treatment period (weeks 8–16), a statistical significant difference was observed between HFHS and HFHS+vit D (weeks 8–16 [g/die]: 2.6±0.3 Control, 2.3±0.4 Control+vit D, 2.8±0.7 HFHS, 2.3±0.7 HFHS+vit D, p<0.05 HFHS *vs* HFHS+vit D; week 1–16 [g/die]: 2.5±0.3 Control, 2.4±0.3 Control+vit D, 2.7±0.6 HFHS, 2.5±0.3 HFHS+vit D).

### Effect of diet manipulation and 1,25 (OH)_2_ vitamin D supplementation on serum 25-OH vitamin D level

**Fig 2:**
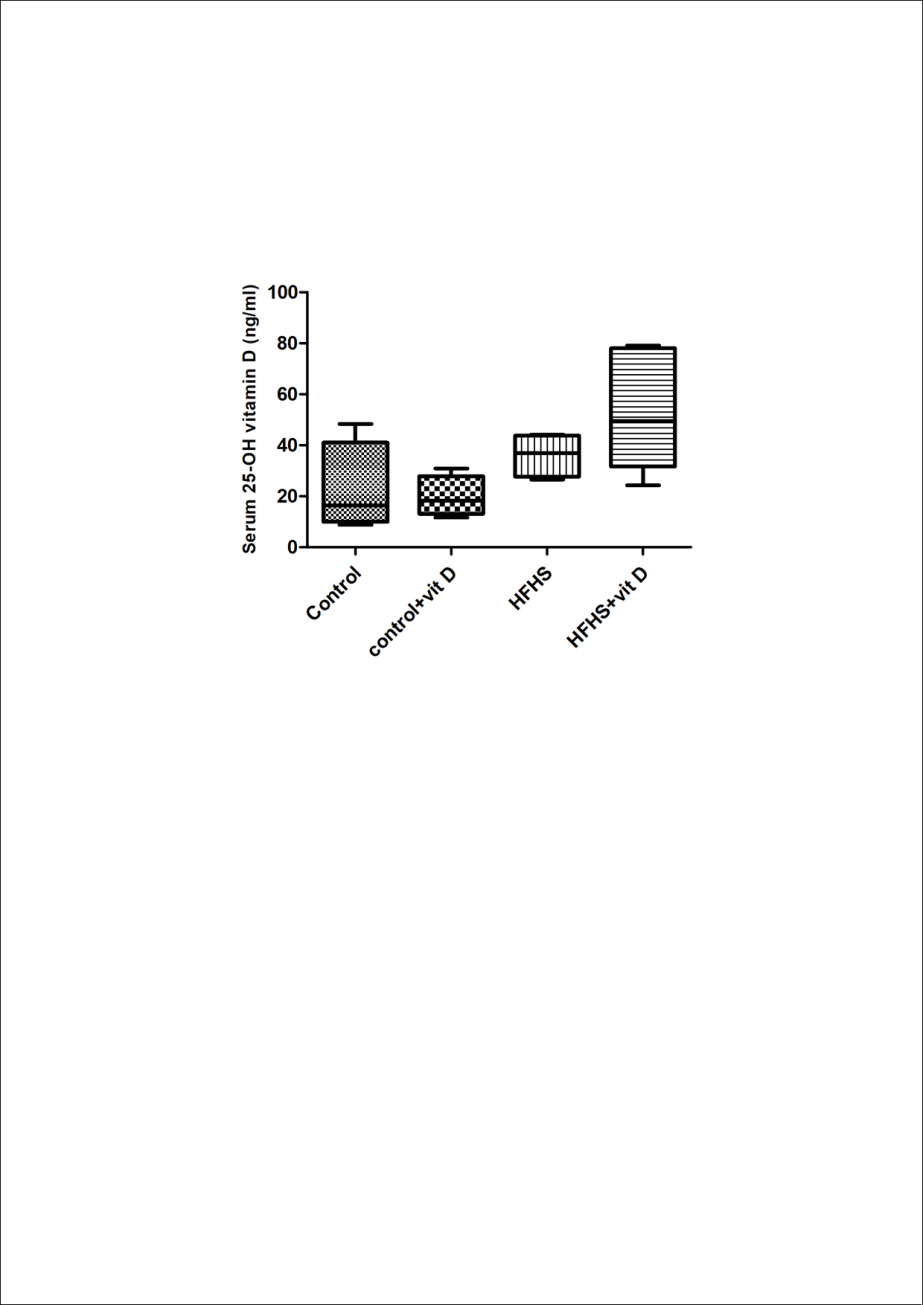
Effects of diet manipulation and 1,25 (OH)_2_ vitamin D supplementation on serum 25-OH vitamin D level. Levels of 25-OH vitamin D were measured by ELISA in the serum of 4–6 randomly selected animals per group. Data are expressed by medians and interquartile range. Statistical analysis was performed with Kruskal–Wallis test with Dunn’s post hoc test.

Serum level of 25-OH vitamin D, the most stable metabolite, was not affected either by diet manipulation or by 1,25 (OH)_2_ vitamin D supplementation. As shown in the Fig 2 no statistically significant differences were observed among the groups, albeit an upward trend was evident for the HFHS+vit D group compared to the HFHS group.

### Effects of diet manipulation and 1,25 (OH)_2_ vitamin D supplementation on oral glucose tolerance test, HOMA index and lipid profile

**Fig 3:**
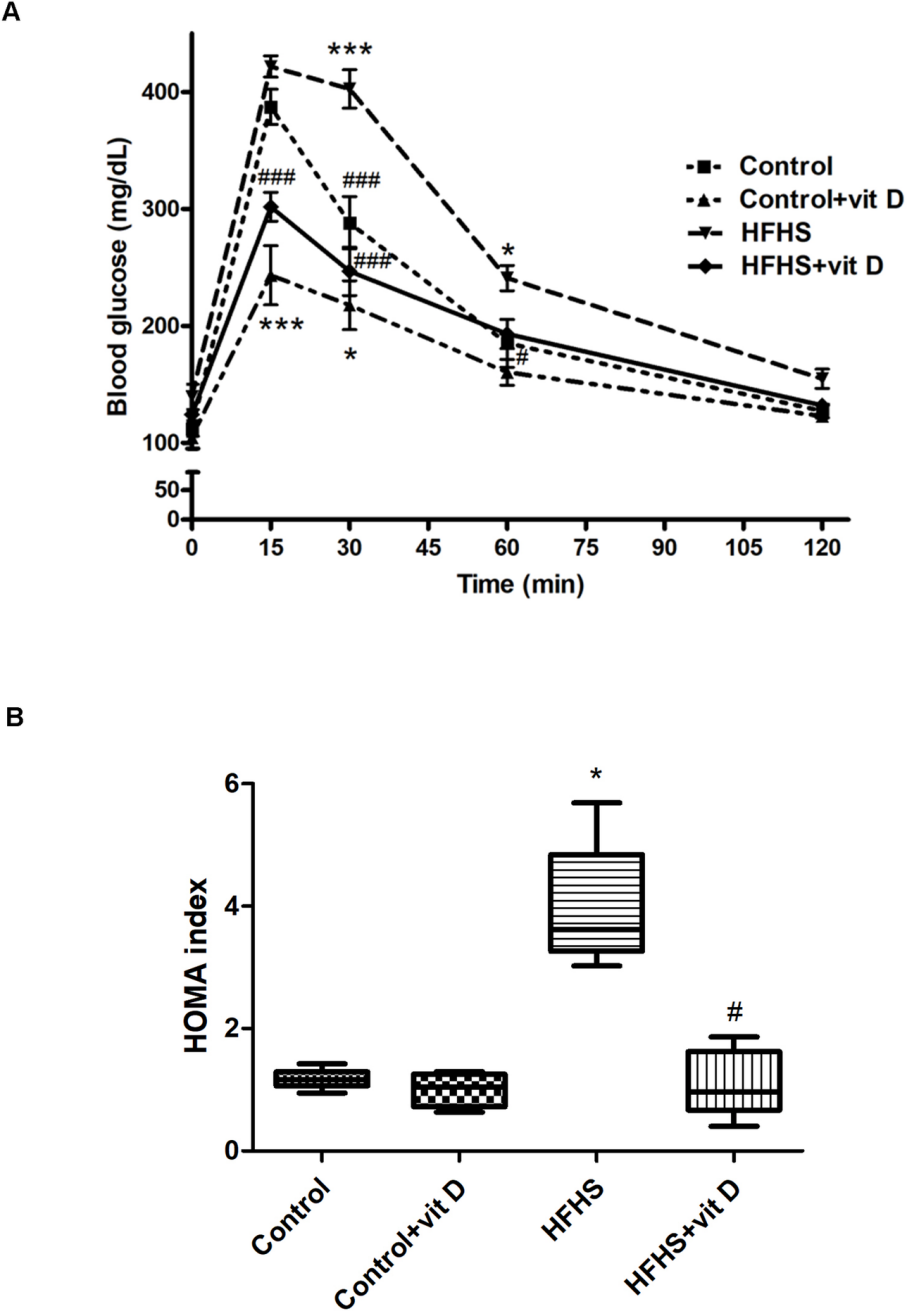
Effects of diet manipulation and 1,25 (OH)_2_ vitamin D supplementation on oral glucose tolerance test and HOMA index measured at the end of the experimental protocol (week 16) in fasted animals. Panel A. Oral glucose tolerance test **(**OGTT). Values are means ± S.E.M. of 6–10 animals per group. Statistical analysis was performed with Two-way analysis of variance with Bonferroni's post-hoc test. *p<0.05, ***p<0.001 *vs* Control; ^#^p<0.05, ^##^p<0.01, ^###^p<0.001 *vs* HFHS. Panel B. HOMA index. Data are expressed by medians and interquartile range of 4–6 animals randomly selected per group. Statistical analysis was performed with Kruskal–Wallis test with Dunn’s post hoc test. *p<0.05 *vs* Control; ^#^p<0.05 *vs* HFHS.

A significant impairment of the glucose tolerance was evident during OGTT in HFHS animals in comparison with control animals (Fig 3A). Noteworthy, animals treated with vitamin D not only showed a significant improvement of the glucose tolerance, but were also unable to reach the glucose plasmatic peak induced by the glucose load. Indeed, in both groups of animals treated with vitamin D (Control+vit D and HFHS+vit D) the level of glycemia measured after the glucose load was significantly lower in comparison with the control group (p<0.001).

Accordingly, the HFHS group showed a three-fold (p<0.05) increase in HOMA-IR index in comparison to the control group, while vitamin D treatment was associated with a significant improvement of this parameter (Fig 3B).

Neither HFHS diet neither vitamin D treatment significantly affected the plasma lipid profile (except for LDL cholesterol, HFHS *vs* Control: p<0.05; LDL cholesterol [mg/dl]: 36.43±5.51 Control, 43.41±8.20 Control+vit D, 59.11±4.16 HFHS, 49.41±7.19 HFHS+vit D; total cholesterol [mg/dl]: 98±9.49 Control, 111.97±9.06 Control+vit D, 124.5±4.81 HFHS, 102.8±11.44 HFHS+vit D; plasma triglycerides [mg/dl]: 51.5±6.71 Control, 52.07±3.04 Control+vit D, 64.08±3.78 HFHS, 47.27±7.22 HFHS+vit D).

### Effects of dietary manipulation and 1,25 (OH)_2_ vitamin D supplementation on insulin signaling transduction in the mouse gastrocnemious

**Fig 4:**
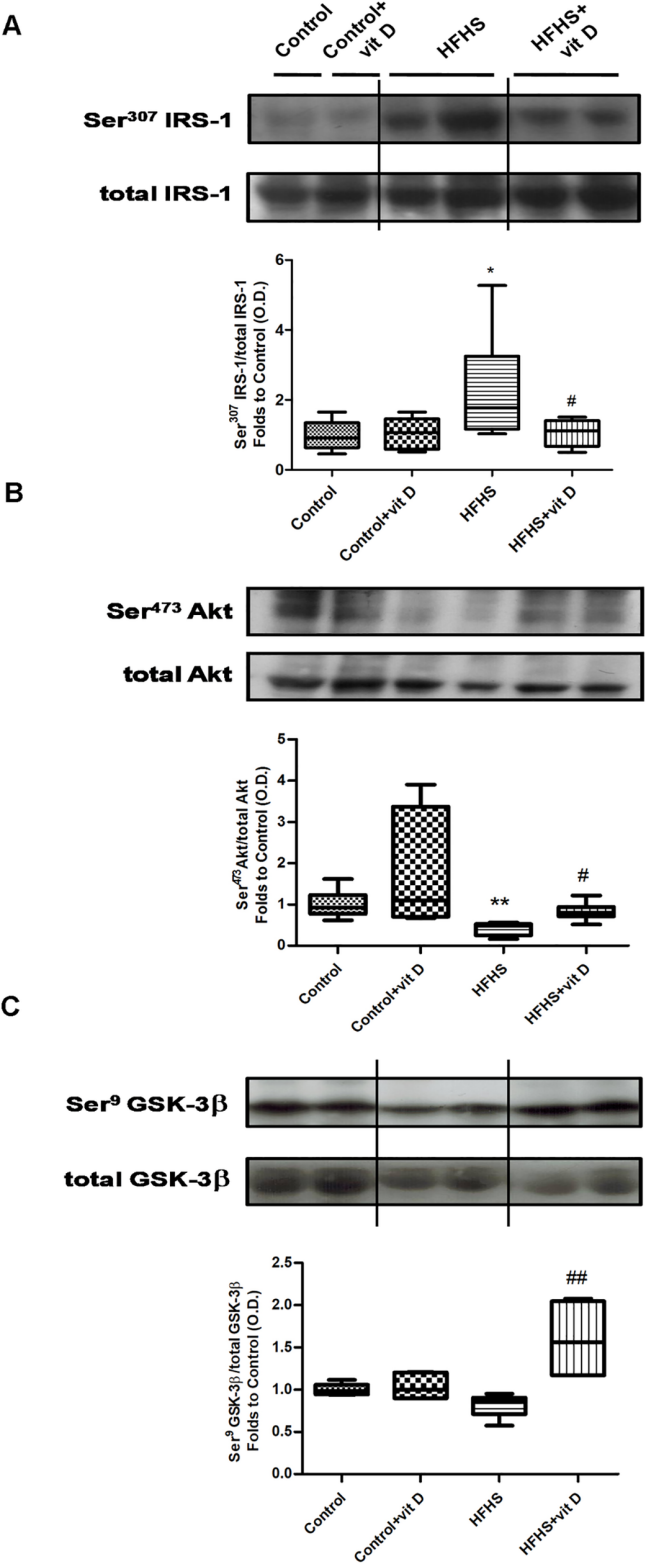
Effects of diet manipulation and 1,25 (OH)_2_ vitamin D supplementation on insulin signaling transduction in the mouse gastrocnemious. The expression of total IRS-1 protein and its Ser^307^ phosphorylation (panel A), total Akt protein and its Ser^473^ phosphorylation (panel B), and total GSK-3β protein and its Ser^9^ phosphorylation (panel C) were analyzed by Western blot on gastrocnemious homogenates of animals fed a control diet or HFHS diet, with or without vitamin D supplementation (7 μg/kg, 3 times per week). Densitometric analysis of the bands is expressed as relative optical density (O.D.) and normalized using the related control band. The data are expressed by medians and interquartile range of 5–8 randomly selected animals per group. Statistical analysis was performed with Kruskal–Wallis test with Dunn’s post hoc test. *p<0.05, **p<0.01 *vs* Control; #p<0.05, ##p<0.01 *vs* HFHS.

As shown in Fig 4, a significant impairment of the insulin signaling pathway was detected in the gastrocnemious muscle of animals fed HFHS diet. In fact, a significant increase of Ser^307^ phosphorylation of IRS-1 (Panel A) was detected in the HFHS group and this effect was associated to a decrease of Ser^473^ phosphorylation of Akt (Panel B), a well known marker of insulin sensitivity. All these deleterious effects evoked by the diet were modified by vitamin D administration (HFHS+vit D *vs* HFHS: p<0.05) that was able to blunt the Ser^307^ phosphorylation of IRS-1 and increase the Ser^473^ phosphorylation of Akt.

In addition, a significant increase of the inactive form of GSK-3β (phosphorylated in Ser^9^), a downstream target of Akt insulin signaling, was evident in HFHS animals treated with vitamin D (Panel C, HFHS+vit D *vs* HFHS: p<0.01).

### Effects of dietary manipulation and 1,25 (OH)_2_ vitamin D supplementation on skeletal muscle inflammation

**Fig 5:**
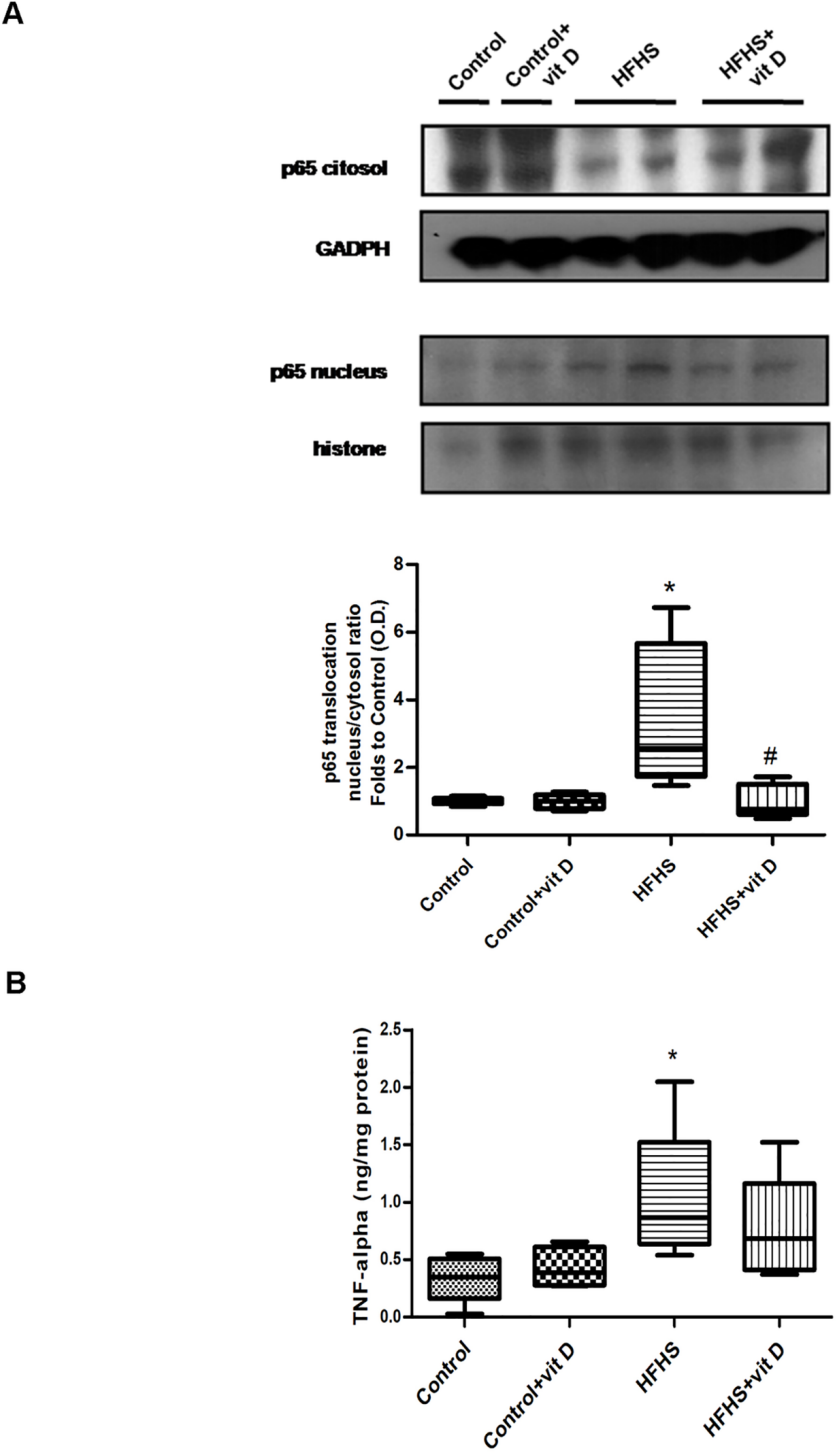
Effects of diet manipulation and 1,25 (OH)_2_ vitamin D supplementation on NF-κB activation (panel A) and TNF-alpha levels (panel B) in the mouse gastrocnemious. (A) Representative western blotting analysis for the expression of NF-κB p65 subunit. Protein expression was analyzed by Western blot on cytosol and nucleus homogenates of gastrocnemious from animals fed a control diet or HFHS diet with or without vitamin D supplementation (7 μg/kg, 3 times per week). Densitometric analysis of the bands is expressed as relative optical density (O.D.) corrected for the GADPH (cytosol) or histone (nucleus) contents, and normalized using the related control band. NF-κB p65 subunit translocation was expressed as nucleus/cytosol ratio normalized using the related control band. (B) TNF-alpha levels were measured by ELISA in the mouse gastrocnemious homogenates. The data are expressed by medians and interquartile range) of 5–6 randomly selected animals per group. Statistical analysis was performed with Kruskal–Wallis test with Dunn’s post hoc test. *p<0.05 *vs* Control; #p<0.05 *vs* HFHS.

In order to evaluate the effects of vitamin D against diet-induced inflammation, the activation of NF-κB was assessed in gastrocnemious homogenates (Fig 5).

As shown by the immunoblotting analyses, a significant (p<0.05) increase in the translocation of the NF-κB subunit p65 from the cytosolic to the nuclear fraction of tissue extracts was detected in the HFHS group compared with control group, while NF-κB activation was significantly reduced in HFHS animals treated with vitamin D (p<0.05 *vs* HFHS).

Accordingly, a marked increase of TNF-alpha, the main pro-inflammatory cytokine involved in insulin resistance development, was detected in the gastrocnemious of HFHS animals (p<0.05 *vs* Control). Vitamin D administration was associated with a weak (although not significant: p>0.05 *vs* HFHS) decrease of TNF-alpha level.

### Effects of dietary manipulation and 1,25 (OH)_2_ vitamin D supplementation on skeletal muscle lipid accumulation

**Fig 6:**
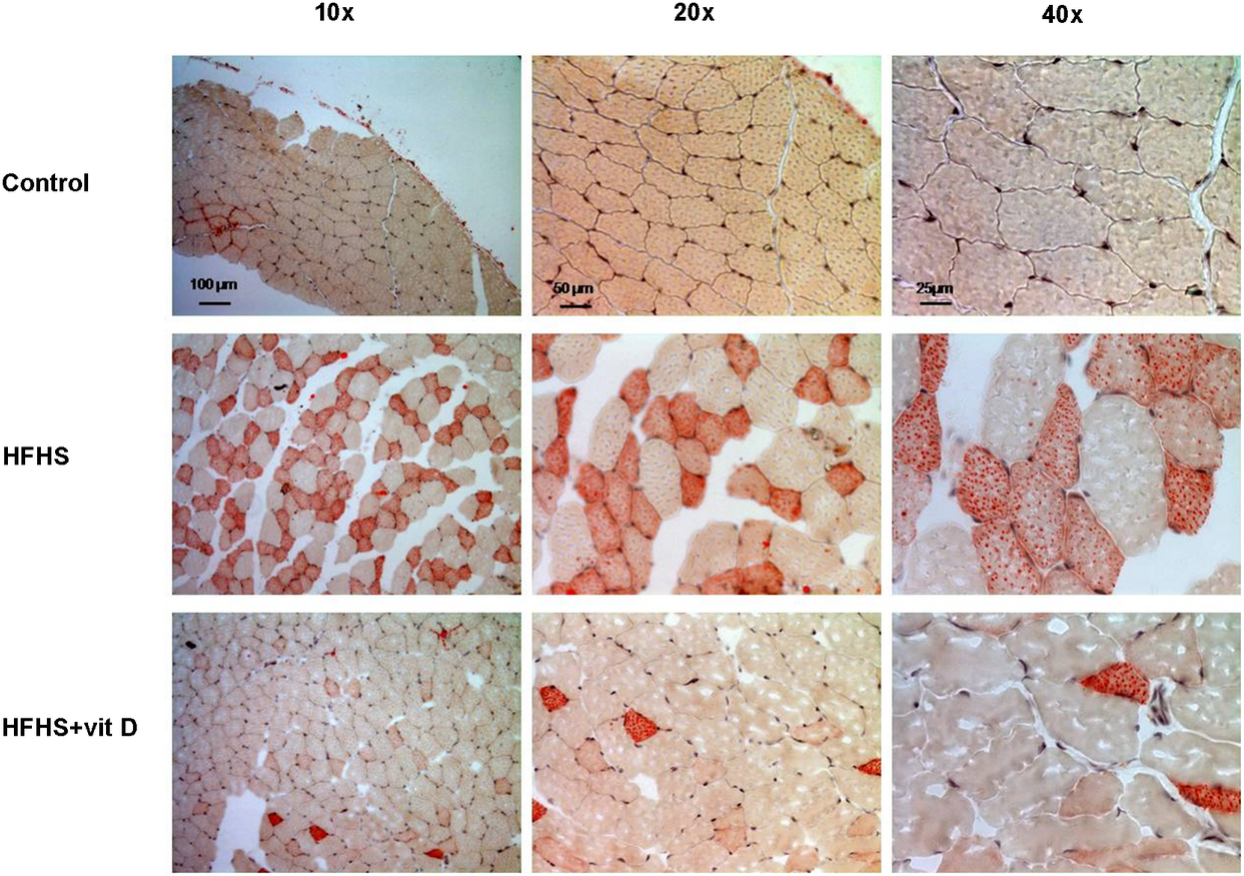
Effects of diet manipulation and 1,25 (OH)_2_ vitamin D supplementation on lipid accumulation in the mouse gastrocnemious. Representative photomicrographs (10x, 20x, 40x magnification) of Oil Red staining on gastrocnemious sections from animals fed a control diet or HFHS diet with or without vitamin D supplementation (7 μg/kg, 3 times per week).

As shown in the representative picture (Fig 6), fat accumulation in skeletal muscle was evident on gastrocnemious sections of HFHS mice, while a clear reduction was observed in animals treated with vitamin D (Fig 6). This data suggested that vitamin D administration reduced the diet-induced myosteatosis in the gastrocnemious muscle of HFHS animals.

**Fig 7:**
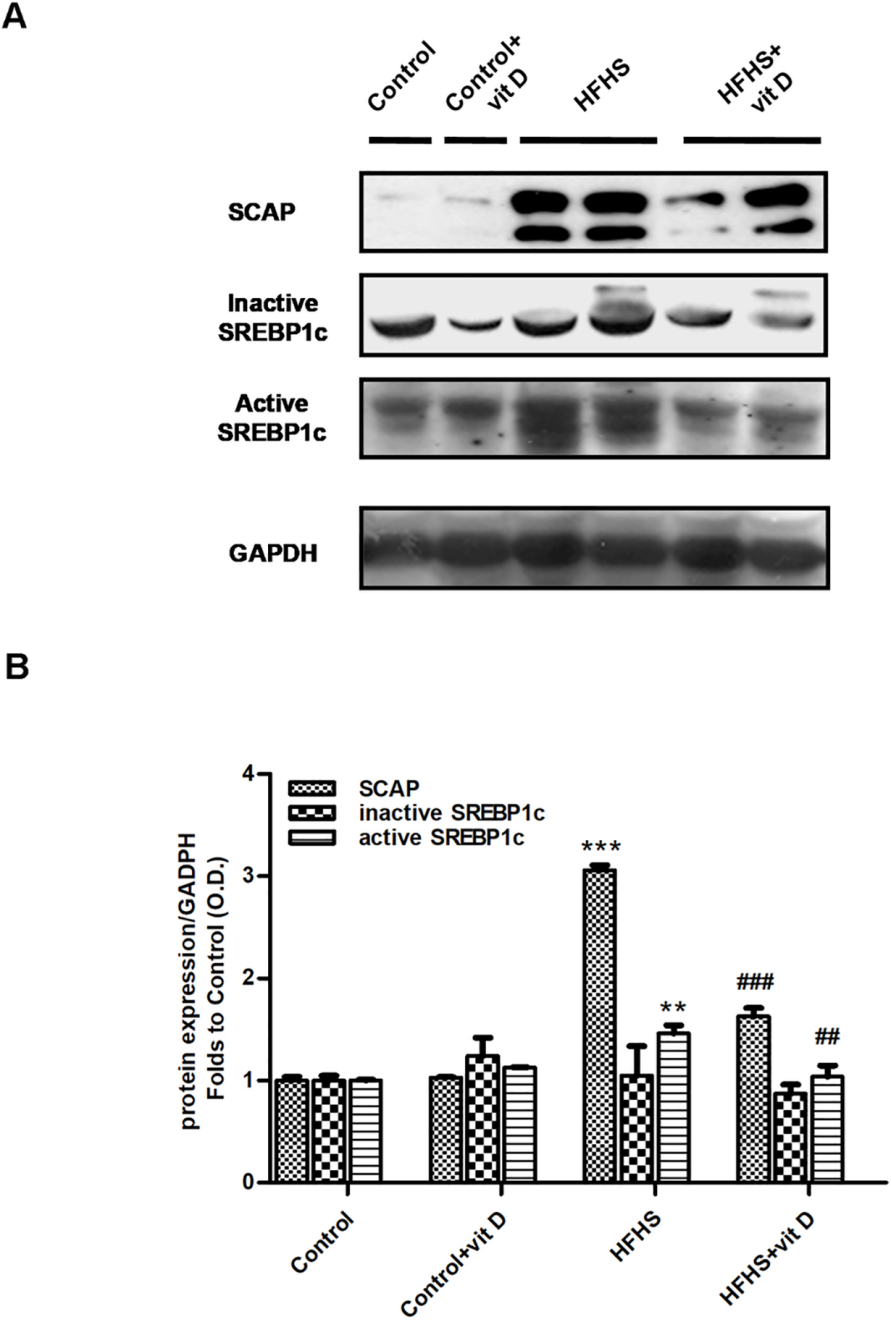
Effects of diet manipulation and 1,25 (OH)_2_ vitamin D supplementation on SCAP/SREBP pathway activation in the mouse gastrocnemious. (A) Representative Western blotting analysis for the expression of SCAP and active/inactive SREBP1c. Protein expression was evaluated on gastrocnemious homogenates of animals fed a control diet or HFHS diet with or without vitamin D supplementation (7 μg/kg, 3 times per week). (B) Densitometric analysis of the bands is expressed as relative optical density (O.D.), corrected for the GADPH contents, and normalized using the related control band. The data are means ± S.E.M. of 6–8 randomly selected animals per group. Statistical analysis was performed by One-way analysis of variance with Bonferroni's post-hoc test. **p<0.01, ***p<0.001 *vs* Control; ^##^p<0.01, ^###^p<0.001 *vs* HFHS.

In order to identify the source of fat accumulation, the activation of the SCAP/SREBP1c lipogenic pathway was assessed by immunoblotting on gastrocnemious homogenates from mice fed a standard or HFHS diet with or without vitamin D treatment (Fig 7).

A three-fold increase of SCAP expression was detected in HFHS animals compared to control mice (HFHS *vs* Control: p<0.001). Accordingly, an higher expression of the active form of SREBP1c was detected in animals fed HFHS diet compared to control mice (HFHS *vs* Control: p<0.01). No differences were detected for the inactive form of SREBP1c among the different groups of animals.

### Effects of dietary manipulation and 1,25 (OH)_2_ vitamin D supplementation on AGEs accumulation and RAGE expression in the mouse gastrocnemious

**Fig 8:**
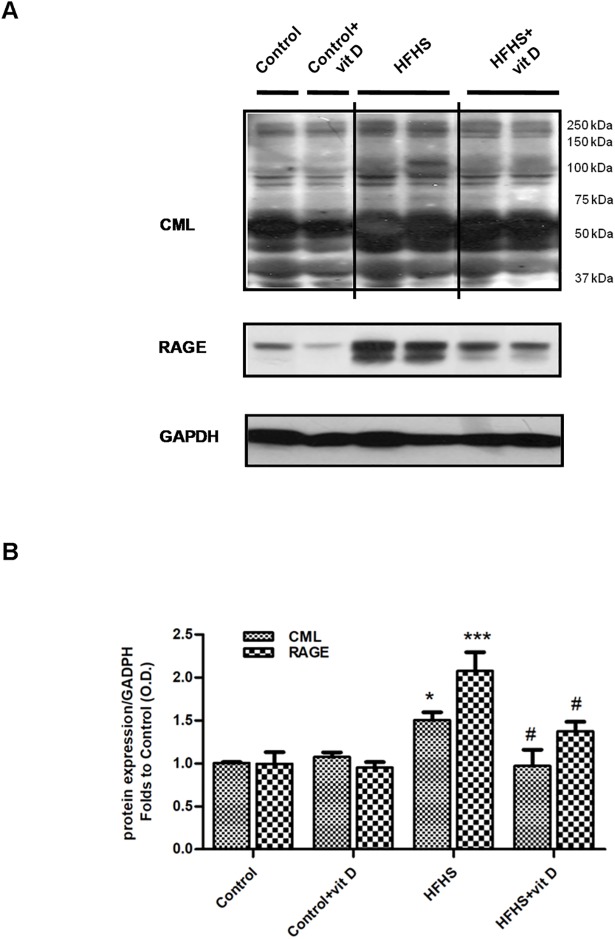
Effects of diet manipulation and 1,25 (OH)_2_ vitamin D supplementation on levels of CML protein adducts and RAGE expression in the mouse gastrocnemious. (A) Representative Western blotting analysis for CML-modified proteins and RAGE expression. Protein expression was evaluated on gastrocnemious homogenates of animals fed a control diet or HFHS diet with or without vitamin D supplementation (7 μg/kg, 3 times per week). (B) Densitometric analysis of the bands is expressed as relative optical density (O.D.), corrected for the GADPH contents, and normalized using the related control band. The data aremeans ± S.E.M. of 6–9 randomly selected animals per group. Statistical analysis was performed by One-way analysis of variance with Bonferroni's post-hoc test. *p<0.05, ***p<0.001 *vs* Control; #p<0.05 *vs* HFHS.

Western blotting analysis of gastrocnemious homogenates from HFHS animals showed increased levels of proteins modified by CML and increased expression of the AGE receptor, RAGE, in comparison with control animals. Moreover, the HFHS+vit D group showed levels of CML protein adducts and RAGE expression significantly lower compared to those of HFHS mice (Fig 8, HFHS+vit D *vs* HFHS: p<0.05). Briefly, vitamin D administration reduced the diet-induced accumulation of AGEs and RAGE (over)expression in the gastrocnemious muscle of HFHS animals.

## Discussion

The present study showed that administration of vitamin D to diet-induced obese mice halted the progression in body weight gain, hyperglycemia and hyperinsulinemia induced by diet. In keeping with previous studies [23, 24], here we used the active vitamin D form, the 1,25 (OH)_2_ vitamin D, at the dose of 7 μg/kg i.p. three times/week, which did not affect the serum level of the reservoir form of vitamin D (the 25-OH vitamin D). Besides, the serum level here recorded was significantly below the threshold of toxicity. In fact, serum levels of 25-OH vitamin D above 150 ng/ml have been demonstrated to be required to induce hypercalcemia and hyperphosphatemia [25].

Although these observations are in keeping with previous studies on the effects of vitamin D in animals fed with hypercaloric diets [13, 14, 26], much is still unknown about the specific mechanism(s) that may contribute to the observed beneficial effects.

In keeping with previous studies [27, 28], here we adopted an hypercaloric diet enriched not only in fats but also in sugars. Indeed, sugar consumption is dramatically increased in the last years and several epidemiological studies showed a correlation between the increase of sugar intake and the development of obesity or the onset of metabolic disturbances including, hyperglycemia, and insulin resistance. In particular, excessive sugar consumption is associated to an increased ectopic lipid deposition, above all in liver and skeletal muscle [29, 30]. This effect has been demonstrated to result from the activation of the lipogenesis pathway SCAP-SREBP1c [16]. Interestingly, several lines of evidence showed that a crucial role in the SREBP activation could be attributed to the action of AGEs, of which the sugars represent a substantial source [31]. Thus, the model herein reported allowed us to better mimic the damage induced by a typical human hypercaloric diet containing both lipids and sugars and investigate the mechanism underlying the vitamin effects, especially against AGEs accumulation and myosteatosis.

The data here reported showed, for the first time, that the protection exerted by vitamin D against diet-induced metabolic derangements is due, at least in part, to interference with selective signaling pathways within the skeletal muscle, which is the major site of glucose disposal and, thus, exerts a key role in regulating whole body glucose homeostasis. Our results demonstrated that vitamin D significantly improved muscle insulin resistance, one of the main defects leading to type 2 diabetes mellitus [32], by counteracting the diet-induced reduction in efficiency of the insulin pathway, shown by the impaired phosphorylation of IRS-1 protein as well as of the downstream key insulin signalling molecules, Akt, and the Akt substrate GSK-3β. This effect is known to be associated with an increased expression and translocation to the cell membrane of the predominant glucose transporter in the skeletal muscle, the glucose transporter 4 (GLUT4). Our *in vivo* data are in keeping with previously published *in vitro* results showing that vitamin D improves the insulin signalling pathway [33] and up-regulates the GLUT4 translocation [34], thus proving that both these effects are essential for maintenance of glucose metabolism.

However, so far, the effects of vitamin D on molecular mechanism(s) affecting insulin sensitivity have been scarcely investigated in *in vivo* models [35]. A previous study demonstrated that vitamin D treatment enhanced IRS-1 transcription in muscle but not in liver and adipose tissues of mice exposed to hypercaloric diet [36]. More recently, Chen et al. generated skeletal muscle specific vitamin D receptor-null mice demonstrating that vitamin D signalling deficiency in the skeletal muscle significantly contributed to the development of insulin resistance and glucose intolerance [37]. It is well known that chronic exposure to pro-inflammatory mediators stimulates the activation of cytokine molecular pathways which ultimately block the insulin signalling. In particular, TNF-alpha, which is highly expressed in skeletal muscle [38], adipose tissue [39], and plasma [40], is one of the main mediators of the cross-talk mechanism linking obesity to insulin resistance [41]. Here we confirmed an overexpression of TNF-alpha within the skeletal muscle of obese/insulin resistant mice and, most notably, we demonstrated that vitamin D supplementation reported local TNF-alpha levels back to values similar to those recorded in control mice. TNF-alpha is a well-known downstream target of the inflammatory pathway regulated by NF-κB, which is widely activated in the skeletal muscle of animals subjected to an hypercaloric diet [42]. Vitamin D has been previously demonstrated to suppress the NF-κB activation and therefore the transcription of its downstream targets [43–47] in different cell types, including muscle cells. We, thus, decided to investigate whether or not the modulation of NF-κB inflammatory pathway is involved in its beneficial effects against insulin resistance. Here, we show, for the first time, that vitamin D reverted the diet-induced increase in the nuclear translocation of NF-κB p65 in the mouse gastrocnemious. The reduction of NF-κB activation by vitamin D most likely accounts for the observed reduction in the local levels of TNF-alpha, thus resulting in improvement of the insulin signalling pathway. At the same time, the preserved functionality of the insulin pathway may contribute to further inhibit the activation of NF-κB. In fact, specific inhibition of GSK-3β has been shown to directly inhibit NF-κB-dependent gene transcription, probably due to the presence of four phosphorylation sites for the action of GSK-3β on the p65 subunit of NF-κB [48]. Besides, genetic deletion of GSK-3β abrogates activation of IkappaBalpha kinase, which is required to induce NF-κB nuclear translocation [49]. Thus, the vitamin D-induced inhibition of GSK-3β (here shown in terms of increased Ser^9^ phosphorylation) is suggestive not only of improved insulin signalling downstream of IRS-1 but also of reinforced inhibition of NF-κB nuclear translocation. We may speculate that downregulated GSK-3β activity by vitamin D supplementation could lead to less NF-κB activation, decreasing cytokine production and thus, forming a feed-forward mechanism and further reducing the development of insulin resistance. The reduction in weight gain exerted by vitamin D supplementation represents another important effect contributing to preserve local and systemic insulin sensitivity, being obesity a main causative factor for the development of insulin resistance. Notably, recent experimental evidence suggested that obesity is not *per se* the driver of insulin resistance, but rather the accumulation of intracellular lipid metabolites is the key trigger leading to insulin resistance [50]. Our results convincingly supported the hypothesis that vitamin D may significantly counteract the diet-induced myosteatosis. Indeed, we found a significant increase of lipid accumulation in the skeletal muscle of mice treated for 16 weeks with the hypercaloric diet and this deleterious effect was reduced by vitamin D supplementation. As we previously demonstrated [15], a mechanism underlying the increase of muscle lipogenesis is SREBP1c activation, which contributes to myosteatosis development. SREBP1c is a transcription factor that regulates lipid homeostasis, being a potent activator of the fatty acid synthesis [51]. The primary modulator of SREBP1c activity is SCAP, which is both an escort for SREBP1c and a sensor of sterols. When cells need sterols, SCAP transports SREBP1c from the endoplasmic reticulum to the Golgi apparatus, where it is cleaved by two proteases. The cleaved form of SREBP1c is able to enter in the nucleus, binds to the sterol-regulatory elements, and increase the transcription of target genes. Interestingly, we showed that treatment with vitamin D reversed the diet-induced increase of SCAP and active SREBP1c expression in mouse gastrocnemious, thus demonstrating, for the first time, that the inhibition of the SREBP1c/SCAP lipogenic pathway is a key molecular event leading to vitamin D-dependent reduction of myosteatosis. Very recently, we also documented that the AGEs accumulation, the co-localisation between AGEs and SCAP-(hyper)expressing cells (suggestive for SCAP glycosylation) and hyperinsulinemia combine to cause SREBP1c activation in muscle fibers [15, 16]. Therefore, here we investigated whether or not the interference with the AGE/RAGE pathway may underlie the vitamin D effects within the skeletal muscle. Our results convincingly demonstrated that vitamin D administration evoked a significant reduction of one of the major AGEs, the CML protein adducts. This effect, likely due to an improvement of insulin sensitivity exerted by vitamin D, could determine a reduction of SCAP glycosylation and, accordingly, of SREBP1c/SCAP lipogenic pathway, thus contributing to the beneficial effects evoked by vitamin D against myosteatosis.

Vitamin D supplementation was also associated with a robust decrease in the expression of the AGEs receptor, RAGE. As the expression of both RAGE and SREBP1c can be regulated by NF-κB, being NF-κB-like binding sites in their proximal promoters [52, 53], we cannot rule out the hypothesis that the beneficial effects of vitamin D on these molecular pathways are due to its interference with NF-κB nuclear translocation.

Overall, our results do not allow us either to speculate on direct effects of vitamin D on myosteatosis or to identify which factors had the greatest effect on the modulation of the SREBP1c/SCAP lipogenic pathway. Moreover, our findings could be secondary to vitamin D-induced changes in other organs, liver included. The liver has been already reported to be a vitamin D target, with vitamin D ameliorating hepatic glucose and lipid metabolism abnormalities [54]. Moreover, 1,25 (OH)_2_ vitamin D has been demonstrated to prevent liver fibrosis [55] and to protect the liver structure of mice under high fat diet [56]. However, the hypothesis of a direct effect of Vitamin D on skeletal muscle is supported by previous studies demonstrating the expression of vitamin D receptor in the muscle [57]. This finding was also recently confirmed by the demonstration of the pleiotropic effects of the vitamin D metabolome on muscle strength and function [58].

Further investigation would be required to better elucidate the clinical relevance of the findings here reported on insulin sensitivity. Indeed, despite a large evidence from epidemiological data on a negative relationship between vitamin D levels and insulin sensitivity, so far it is still unclear whether this association reflects a causal relationship or not. Unfortunately, data obtained from human longitudinal studies on vitamin D supplementation are contradictory [59–61]. The inconsistent results suggest that the dose, as well as the genetic background and baseline characteristics of the study population, may affect the efficacy of vitamin D supplementation. In conclusion, our findings show for the first time that supplementation with vitamin D exerts beneficial effects against the metabolic derangements induced by an hypercaloric diet through significant improvements of the signalling events traditionally associated to the development of insulin resistance and myosteatosis within the skeletal muscle.

## Supporting information

S1 Table Diet compositions. (DOC)
