# Supplementary material for: Effects of vitamin D on insulin resistance and myosteatosis in diet-induced obese mice
Source: PLoS One. 2018 Jan 17;13(1):e0189707. doi: 10.1371/journal.pone.0189707 (PMC5771572; doi:10.1371/journal.pone.0189707)
Supplement: S1 Table — (DOC) [file pone.0189707.s001.doc]

**S 1 Table. Diet compositions**

| **Ingredient** | **U** | **Control diet** | **HFHS diet** |
| --- | --- | --- | --- |
| ***Casein*** | % | 20.7 | 26.00 |
| ***Corn starch*** | % | 48.82 | 7.77 |
| ***Maltodextrin*** | % | 14.00 | - |
| ***Fructose*** | % | - | 16.5 |
| ***Glucose*** | % | - | 13.5 |
| ***Cellulose*** | % | 5 | 5.7 |
| ***L-Cystine*** | % | 0.25 | 0.30 |
| ***Vitamin premix*** | % | 1.00 | 1.00 |
| ***Mineral premix*** | % | 6.00 | 6.00 |
| ***Lard*** | % | 1.6 | 20.5 |
| ***Soybean oil*** | % | 2.4 | 2.5 |
| ***Proximate contents*** | | | |
| ***Crude protein*** | % | 18.2 | 22.8 |
| ***Crude fat*** | % | 4.1 | 23.1 |
| ***Crude fibre*** | % | 5.0 | 5.7 |
| ***Crude ash*** | % | 5.3 | 5.3 |
| ***Starch*** | % | 47.8 | 7.4 |
| ***Sugar*** | % | 1.1 | 30.9 |
| ***Vitamin A*** | UI/kg | 15,000 | 15,000 |
| ***Vitamin D3*** | UI/kg | 1,500 | 1,500 |
| ***Vitamin E*** | mg/kg | 150 | 150 |
| ***Energy* kcal/kg 3,655 4,580**  ***kcal Protein* % 20 20**  ***kcal Fat*  % 10 45**  ***kcal Carbohydrates* % 70 35** | | | |
